# Vasopressinergic Neurocircuitry Regulating Social Attachment in a Monogamous Species

**DOI:** 10.3389/fendo.2017.00265

**Published:** 2017-10-11

**Authors:** Maria C. Tickerhoof, Adam S. Smith

**Affiliations:** ^1^Department of Pharmacology and Toxicology, School of Pharmacy, University of Kansas, Lawrence, KS, United States

**Keywords:** vasopressin, prairie vole, pair bond, partner preference, aggression, parental care

## Abstract

The prairie vole (*Microtus ochrogaster*) is a socially monogamous rodent species that forms a lasting connection between mates, known as a pair bond. The pair bond is primarily characterized by three distinct behaviors: partner preference, selective aggression, and biparental care of the young. The presence of these behaviors in the prairie vole and their absence in closely related non-monogamous species makes the prairie vole an important model of social relationships and facilitates the study of the neurobiological mechanisms of social affiliation and attachment. The nona-peptide arginine-vasopressin (AVP) is an important neuromodulator of social behavior and has been implicated in the regulation of the pair bond-related behaviors of the prairie vole, through activation of the AVP receptor subtype 1a (AVPR1a). Modulation of AVPR1a activity in different regions of the prairie vole brain impacts pair bond behavior, suggesting a role of AVP in neurocircuitry responsible for the regulation of social attachment. This review will discuss findings that have suggested the role of AVP in regulation of the pair bond-related behaviors of the prairie vole and the specific brain regions through which AVP acts to impact these unique behaviors.

## Introduction

The prairie vole (*Microtus ochrogaster*) is a small, mouse-sized rodent native to the Midwest region of the United States. This species performs behaviors related to social monogamy, a trait seen in fewer than 5% of mammalian species (1). This social exclusivity is beneficial in areas where populations of animals are spread out such as in the vast Midwest grasslands, as it may be difficult to encounter potential new mates. In fact, opposite-sex pairs of prairie voles are caught together more frequently in areas where the population is less dense (2). The pair bond is a unique, strong affiliative connection between mates of a socially monogamous species. This bond is characterized primarily by behavioral and physiological hallmarks, including preference for a social partner over unfamiliar conspecifics, selective aggression toward intruding conspecifics, nesting together during gestation, and displaying biparental care of offspring, distress, and social-seeking behavior during periods of separation or social loss, and stress alleviation among reunion and consoling behaviors (1, 3). The prairie vole exhibits these distinctive characteristics (2), but closely related non-monogamous species such as the meadow vole (*Microtus pennsylvanicus*) and the montane vole (*M. montanus*) do not (4, 5). Thus, manipulation and description of these behaviors in the prairie vole, as well as cross-species comparison with the meadow vole or montane vole, allows for the study and understanding of the neural mechanisms behind pair bond-related behaviors. For these reasons, the prairie vole has become an attractive model for studying the neurobiological basis of behaviors related to social affiliation and attachment that are not easily modeled in other laboratory species.

Social behavior is regulated by a number of neuromodulators, such as the neuropeptide arginine-vasopressin (AVP). AVP is a key regulator of a number of social behaviors, including social recognition (6, 7), aggression (8, 9), and maternal care (10). Furthermore, it has been determined that the AVP system in the vole brain functions as a neuromodulator of a number of social behaviors critical for the establishment and maintenance of the pair bond between breeding pairs, including partner preference, selective aggression, and paternal care.^1^ AVP primarily acts through three G protein-coupled receptors: the AVP receptor subtype 1a (AVPR1a), subtype 1b (AVPR1b), and AVP receptor type 2 (AVPR2). The distribution of AVPR1a in the prairie vole brain has been well established (11–17). However, the distribution of AVPR1b and AVPR2 in the prairie vole brain has not been characterized, and thus the regulation of social behavior by AVP is primarily attributed to AVPR1a action. Comparative studies of AVPR1a distribution in closely related *Microtus* species have revealed an expression pattern in prairie voles that is similar to the monogamous pine vole (*M. pinetorum*) (11) and distinct from non-monogamous species such as the montane vole and the meadow vole (12–14). As these distribution patterns correlate with unique patterns of social organization and behavior, it has been theorized that expression of AVPR1a has some role in the neurobiological basis of social affiliation and attachment. The first study to investigate the role of central AVP administration in both prairie voles and montane voles found that AVP promoted pair bond-related behavior, namely selective aggression, in the prairie vole, but not in the montane vole (12). In addition, this study found that AVPR1a binding distribution correlated with *avpr1a* mRNA expression levels. Transgenic mice expressing the prairie vole *avpr1a* gene not only display more affiliative behaviors but also have a “prairie vole-like” distribution pattern of AVPR1a that is distinct from that of wildtype mice (18). These findings suggest a relationship between the *avpr1a* gene and AVPR1a protein distribution patterns, thus prompting investigation into the genetic basis of the role of AVPR1a in social behavior.

While both monogamous and non-monogamous vole species share 99% sequence homology of the *avpr1a* gene, prairie vole *avpr1a* is preceded by an extended 5′ flanking microsatellite region that is not present in non-monogamous species (18). It was originally suggested that this microsatellite region contains *cis*-regulatory elements, controlling *avpr1a* gene expression through binding of transcription factors or secondary DNA structure formation, and promotes species differences in AVPR1a expression and social behavior. However, there are incongruences in the reported relationship between microsatellite length and neuronal and behavioral phenotypes (19). For example, variation in microsatellite length has contrasting correlates with variation of AVPR1a binding in several brain regions and bond-related behaviors of the prairie vole (19–25). Furthermore, insertion of either prairie or meadow vole microsatellite structure ahead of the mouse *avpr1a* coding region leads to measurable differences in AVPR1a density in mice brains, though these results do not fully explain the distribution variability observed among vole species (26). Recent work has expanded beyond microsatellite length into other sources of genetic variation. Single-nucleotide polymorphisms within regulatory sequences have been demonstrated to be good predictors of individual differences in cortical AVPR1a expression, sexual fidelity, and spatial use (23, 27, 28), though this is a weaker relationship in wild-caught voles compared with laboratory-reared animals potentially due to increased variation in the developmental environment in wild populations. Further research is needed to explain how *cis*-regulatory variants and other regulatory elements affect individual and species level AVPR1a distribution patterns and social behavior. Nevertheless, the relationship between AVPR1a distribution patterns and pair bond-related behaviors remains, and thus, it is appropriate to investigate the role of AVPR1a in the modulation of such behaviors unique to monogamous species such as the prairie vole. This review will discuss the role of general and site-specific AVPR1a activity in regulation of three key pair bond-related behaviors of the prairie vole: partner preference, selective aggression, and paternal care of the young.

## Partner Preference

One of the defining characteristics of pair bond behavior in prairie voles is a preference for contact with the mate over an opposite-sex stranger, also known as partner preference (2, 29). In the lab, partner preference is measured using a three-chamber social interaction test in which the subject may choose to spend time by itself in a neutral chamber or interact with either the partner or a novel opposite-sex conspecific. If the subject shows a selective preference for contact with the partner rather than with the stranger during a 3-h assessment period, it is determined that a partner preference has been established (30). Male prairie voles will establish a partner preference after 24 h of cohabitation with a new mate (29, 31), and females exhibit this behavior as well (30, 32). This partner preference is enduring and lasts for at least 2 weeks of separation from the mate (33, 34). This preference is not infallible, however, and is diminished after 4 weeks of separation from the mate (33) and may be interrupted if the breeding pair is reproductively unsuccessful (35).

**Figure 1:**
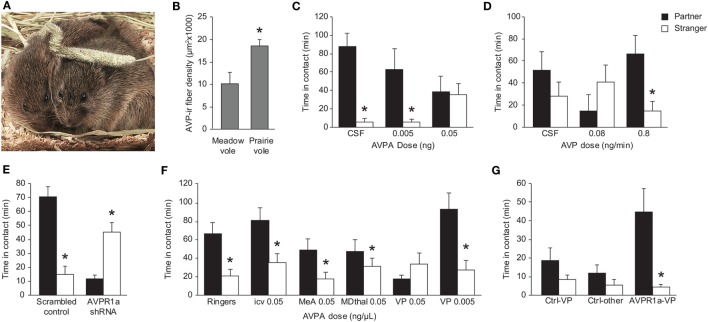
Role of AVP in modulation of prairie vole partner preference. **(A)** A prairie vole breeding pair in direct side-by-side contact. Duration of this kind of contact is a typical marker for affiliation in a partner preference test. Photo credit: Charles Badland. **(B)** Male prairie voles have significantly greater AVP-ir fiber density in the LS compared with male meadow voles. *p < 0.05. Females of both species do not show robust AVP-ir fiber density in this region (data not shown). **(C)** Direct administration of AVPA into the LS of male prairie voles prior to a 24-h cohabitation with a sexually receptive female inhibits partner preference formation in a dose-dependent manner. *p < 0.01 versus duration in contact with the partner. **(D)** Direct infusion of AVP into the LS during a 6-h cohabitation with a non-receptive female induces partner preference in a dose-dependent manner. *p < 0.05 versus duration of contact with the partner. **(E)** Male prairie voles receiving a scrambled shRNA in the VP show a preference for the mate, but shRNA knockdown of AVPR1a in the VP leads to a preference for the stranger. *p < 0.05 between contact with the partner versus the stranger. **(F)** Administration of AVPA into the VP of male prairie voles inhibits partner preference formation. This effect is not seen in animals receiving Ringer’s solution, i.c.v. administration of AVPA, administration of AVPA into the medial amygdala or mediodorsal thalamus, or administration of a 10-fold lower dose of AVPA into the VP. *p < 0.05 versus duration of contact with the partner. **(G)** Meadow voles overexpressing AVPR1a in the VP show a preference for the partner over the stranger following 24-h cohabitation with a sexually receptive female. This effect is not seen in control animals or stereotactic misses. *p < 0.01 versus duration of contact with the stranger. AVP, arginine-vasopressin; AVPA, AVPR1a antagonist; AVPR1a, AVP receptor subtype 1a; i.c.v, intracerebroventricular; ir, immunoreactive; LS, lateral septum; VP, vasopressin. Adapted/reproduced from Wang (39) and Liu et al. (40) with permission from American Psychological Association, Barrett et al. (41) and Lim and Young (42) with permission from Elsevier, and Lim et al. (38) with permission from Nature Publishing Group.

Recognition and affiliation are vital components of partner preference formation, and AVP has been implicated as necessary for these behaviors in mice (6, 7). Winslow and colleagues first demonstrated that male prairie voles will form a partner preference when receiving a central infusion of AVP during a short, non-mated cohabitation but not if receiving a central administration of a selective AVPR1a antagonist (AVPA) immediately prior to a long, mated cohabitation with a female (29). Another study later corroborated these results and revealed that AVP is involved in partner preference formation in female prairie voles as well (32). It has also been suggested that AVP is not only important in the formation of partner preference but also its expression (36). The display of a partner preference is inhibited in male prairie voles that received centrally administered AVPA at the start of a 24-h mated cohabitation or immediately prior to behavioral testing. However, this was not the case for control subjects or those that received AVPA following the 24-h mated cohabitation period in which partner preference was assessed 3 d later, indicating that each administration of AVPA prior to cohabitation and the partner preference test was uniquely responsible for suppression of partner preference behavior. While these studies have suggested both the necessity and sufficiency of AVP in modulating partner preference, they do not suggest which regions of the brain may be involved in AVP-mediated partner preference neurocircuitry. There are a number of regions within the prairie vole brain with high levels of expression of AVPR1a that have been quantified (11–16). Of these regions, AVP signaling in the lateral septum (LS) has been thought to modulate social behavior and organization, and shows different AVPR1a binding levels between monogamous and non-monogamous vole species (11). In addition, the ventral pallidum (VP) shows differential AVPR1a binding between monogamous and non-monogamous vole species, and AVPR1a in this region promotes partner preference (37, 38). Thus, the LS and VP have become regions of interest in the study of the role of AVP in the modulation of partner preference behavior (Figure 1).

### Lateral Septum

Activity of AVP in the LS is known to be vital for social recognition in rats and mice (43–46). Gene transfer of prairie vole AVPR1a in the septum of the rat brain improves recognition of familiar juveniles and promotes more active social interaction behavior (47). Similarly, higher expression of AVPR1a in the LS of male prairie voles is correlated with higher levels of investigatory behavior in response to a novel female (48). In addition, while AVPR1a expression is lower in the LS of male prairie voles compared with male meadow voles, vasopressinergic fiber density in the LS of male prairie voles is significantly higher than that of male meadow voles (39). This suggests that distinct and, potentially, more robust AVP signaling from presynaptic neurons into the LS, and despite lower postsynaptic receptor expression may serve as a mechanism of partner preference behavior. The LS receives vasopressinergic signaling from the bed nucleus of the stria terminalis (BNST) (39, 49), and AVP mRNA expression in the BNST is increased in male prairie voles following a 3-d cohabitation with a female (50). This increase in AVP mRNA expression upstream of the LS is not seen in the non-monogamous meadow vole. Administration of AVPA into the LS of prairie voles during a 24-h mated cohabitation blocks partner preference, and AVP activation of AVPR1a in the LS during a 6-h non-mated cohabitation induces partner preference (40). While similar AVPR1a expression in the LS is observed in both male and female prairie voles (11, 12), AVP innervation into this region is significantly higher in males than it is in females (39, 51, 52). In addition, AVP innervation in the LS of male prairie voles varies over the course of cohabitation with a female, but this effect is not seen in females (53). Taken together, these findings not only implicate a role AVP activity in the LS in partner preference in male prairie voles but also suggest sexual dimorphism in the impact of AVP on partner preference.

### Ventral Pallidum

The VP is located within the basal ganglia and is known to play a role in reward and motivation (54–56). In male prairie voles, it has been determined that AVP signaling and AVPR1a expression in the VP are important in partner preference formation. After 17 h of cohabitation with a non-receptive female, males overexpressing AVPR1a in the VP, but not in the caudate putamen or those treated with LacZ vector, exhibited a significant partner preference (37). A more recent study investigated the role of AVPR1a expression in the VP in modulating partner preference by using short hairpin RNA knockdown of the receptor in this region (41). Male prairie voles with reduced levels of AVPR1a in the VP showed a complete elimination of partner preference behavior. Pharmacological data have also determined that AVPR1a expression in the VP, but not all regions associated with reward or sociosexual neurocircuits, is specifically necessary for partner preference formation. Introduction of AVPA into the VP, but not the medial amygdala or mediodorsal thalamus, prior to a 22-h cohabitation with a receptive female blocked partner preference formation (42). AVPR1a expression in the VP of the prairie vole is considerably higher than in the VP of promiscuous cousins such as the montane vole and the meadow vole (*M. pennsylvanicus*) (13, 38), and overexpression of AVPR1a via virally mediated gene transfer into the VP of the meadow vole induces partner preference formation, a behavior not normally observed in this promiscuous species (38).

Expression of AVPR1a in the VP of female prairie voles varies depending on pair bond and reproductive status. Pair-bonded females exhibit elevated AVPR1a expression in the VP relative to single females, and AVPR1a expression in the VP drops during pregnancy (57). However, AVPR1a levels in the VP are actually elevated immediately following fertilization, and drop back down to pre-pregnancy levels as parturition approaches (58). These findings may also suggest a reason why earlier studies required a higher dose of AVPA in order to manipulate female partner preference behavior (32, 34). Pharmacological treatment of females early in pregnancy may yield different results. However, the behavioral effects of AVP in female prairie voles are not well characterized, and thus further study is needed to understand the behavioral significance of this variation in VP AVPR1a expression.

## Selective Aggression

Sexually naïve prairie voles are highly affiliative and socially tolerant, and will rarely act aggressively to unknown conspecifics. However, after mating has occurred and a pair bond has been established, prairie voles will display robust levels of aggression toward conspecifics entering their territory, but remain highly affiliative to their mates (29, 31, 59–63). This behavior is known as “selective aggression,” which is a type of mate guarding (64, 65) that is specifically a result of mating and the formation of a pair bond. Male prairie voles that have a 24-h cohabitation and mating period with a female will display selective aggression, but prairie voles cohabitating with a same-sex conspecific or for a brief time with a female without mating will not (31, 59). In addition, the promiscuous montane vole, which does not form pair bonds, does not exhibit selective aggression (4). This selective aggression behavior is not limited to intruders of the same sex, as pair bonded male prairie voles will attack unknown female strangers (59–61, 63). Similar to other rodent species, female prairie voles are aggressive during pregnancy and following parturition (2). However, female prairie voles stand apart from females of other rodent species in that female prairie voles also display pair bond induced selective aggression, just as males do (66, 67). Similar to partner preference, selective aggression endures even after a week of separation from the mate (29), but is diminished after 4 weeks of separation (33).

Arginine-vasopressin has been suggested to have a role in aggression in the prairie vole. Administration of AVP via intracerebroventricular (i.c.v.) infusion increases aggressive behaviors of sexually experienced and reproductively successful male prairie voles toward unknown conspecifics (12). This effect is not seen in the non-monogamous montane vole. Infusion of AVPA into the lateral ventricles of male prairie voles that had experienced a 24-h mated cohabitation with a female reduces aggressive behaviors to pre-mated levels, suggesting that AVPR1a is the mediator of this effect (29). In addition, i.c.v. administration of AVP induces selective aggression in sexually naïve males. These findings suggest that AVP has a role in not only general aggressive behaviors but the formation of selective aggression as a form of mate guarding. However, central AVPA administration does not reduce aggression in established breeders, suggesting that full-brain modulation of AVPR1a activity is not sufficient to understand the role of AVP in established selectively aggressive behavior. Therefore, it is necessary to investigate the site-specific regulation of selective aggression by AVP in order to understand its role in expression and maintenance of this behavior.

### Anterior Hypothalamus

**Figure 2:**
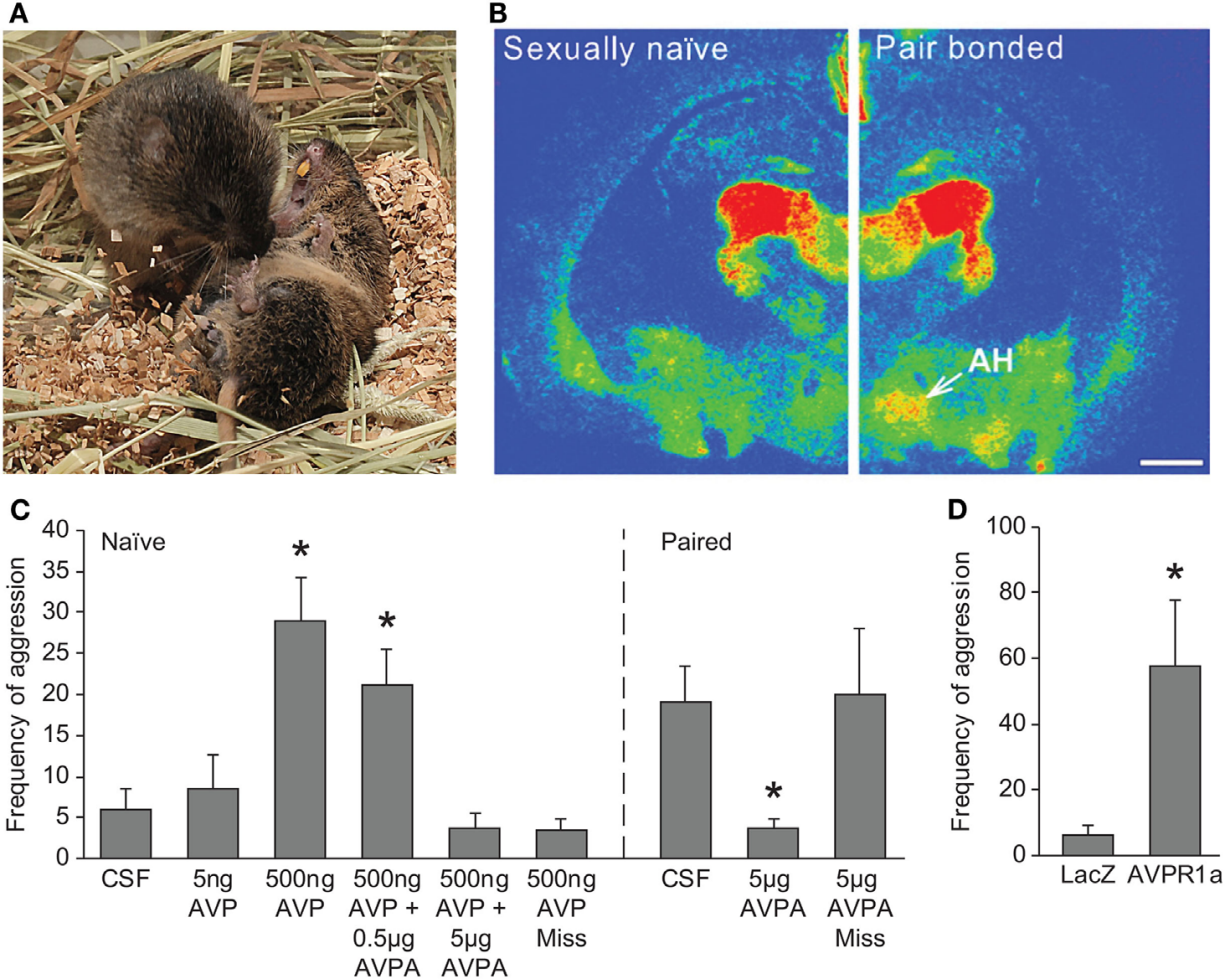
Role of AVP in regulation of selective aggression in prairie voles. **(A)** Aggressive behavior is seen in prairie voles following cohabitation and mating with an opposite-sex animal. Photo credit: Charles Badland. **(B)** A significant increase in AVPR1a binding is observed in the AH of pair-bonded male prairie voles compared with sexually naïve male prairie voles. Scale bar is 1 mm. **(C)** Direct administration of AVP into the AH of sexually naïve male prairie voles induces aggressive behavior, and this effect is blocked by coadministration with AVPA. An increase in aggression is not observed with a stereotactic miss of the AH. Similarly, administration of AVPA into the AH of pair-bonded male prairie voles blocks bond-induced aggressive behavior. *p < 0.05 versus CSF-treated levels. **(D)** Virally mediated gene transfer of AVPR1a into the AH of sexually naïve male prairie voles induces aggressive behavior. *p < 0.05 versus LacZ vector control. AH, anterior hypothalamus; AVP, arginine-vasopressin; AVPA, AVPR1a antagonist; AVPR1a, AVP receptor subtype 1a; CSF, Cerebrospinal fluid. Adapted/reproduced from Gobrogge et al. (61) with permission from Proceedings of the National Academy of Sciences.

Arginine-vasopressin in the anterior hypothalamus (AH) has been found to regulate aggression in hamsters (68, 69). Similarly, recent work by Gobrogge and Wang has established AVPR1a expression in the AH that is modulated by pair bonding, and its activation is important in the regulation of selective aggression in the prairie vole (Figure 2). AVPR1a binding levels are elevated in the AH of pair-bonded male prairie voles relative to non-pair-bonded animals (61). Intriguingly, virally mediated overexpression of AVPR1a in the AH facilitates selective aggression in sexually naïve male prairie voles, suggesting that the increase in receptor expression following pairing may prime the selective aggression observed in male prairie voles. Certainly, exposure of either a female or male stranger, but not the partner, to a pair-bonded male increases neural activation, measured by Fos-immunoreactive (ir) labeling, in the AH (60). This information is supported by the finding that AVP release in the AH is positively correlated with aggression and negatively correlated with affiliation (61). Similar to the i.c.v. studies described above, site-specific AVP administration into the AH induces aggression toward novel females in naïve male prairie voles, and AVPA into the AH of pair-bonded animals reduces aggressive behavior toward stranger females (61). In addition, real-time infusion of AVPA into the AH of a pair-bonded male prairie vole while in the presence of a novel female reduces aggression and increases affiliation, and similar treatment with AVP while in the presence of the partner induces aggression toward the partner (63). These results suggest that AVPR1a in the AH is important not only for the formation of selective aggression but also the decision between aggressive or affiliative behaviors toward the partner or a novel female. Modulation of selective aggression by AH-AVPR1a has not yet been investigated in female prairie voles. Still, similar to a newly pair-bonded male, an increase in AH-AVPR1a has been observed in pregnant pair-bonded female prairie voles relative to pregnant non-pair-bonded female prairie voles (57). This may suggest that the role that AH-AVPR1a plays in regulating selective aggression in female prairie voles may be quite complex, involving both pair bond and pregnancy status.

## Paternal Care

Prairie voles stand out from many mammalian species in parental care of young. Other than nursing, prairie vole fathers are just as involved in the rearing of pups as mothers are, performing parental behaviors such as nest building, licking, grooming, huddling, and pup retrieval (70). In addition, juvenile and sexually naïve male prairie voles display alloparental care of neonates (51, 71–73). While juvenile females will display alloparental behaviors (74), sexually mature but inexperienced females are often neglectful of pups or even infanticidal (71, 72) unless raised to adulthood with the parents (75). For this reason, studies of alloparental care are often performed using male prairie voles. Administration of AVP into the lateral ventricles of sexually naïve male prairie voles does not increase parental behaviors above untreated and vehicle-treated levels (76); this may be the result of a ceiling effect, since sexually naïve male prairie voles are already highly parental. In fact, central administration of AVP can diminish infanticide and promote paternal behavior in male meadow voles that were previously non-paternal, but does not affect paternal behavior in already paternal males (77). However, a high dose of AVPA leads to a higher frequency of pup attack, which is normally a rare behavior. Coadministration of AVPA with an oxytocin receptor (OTR) antagonist significantly reduces parental behaviors as well as further increases the incidence of infanticidal behavior, suggesting that AVPR1a and OTR may work in tandem to promote alloparental behavior.

### Lateral Septum

**Figure 3:**
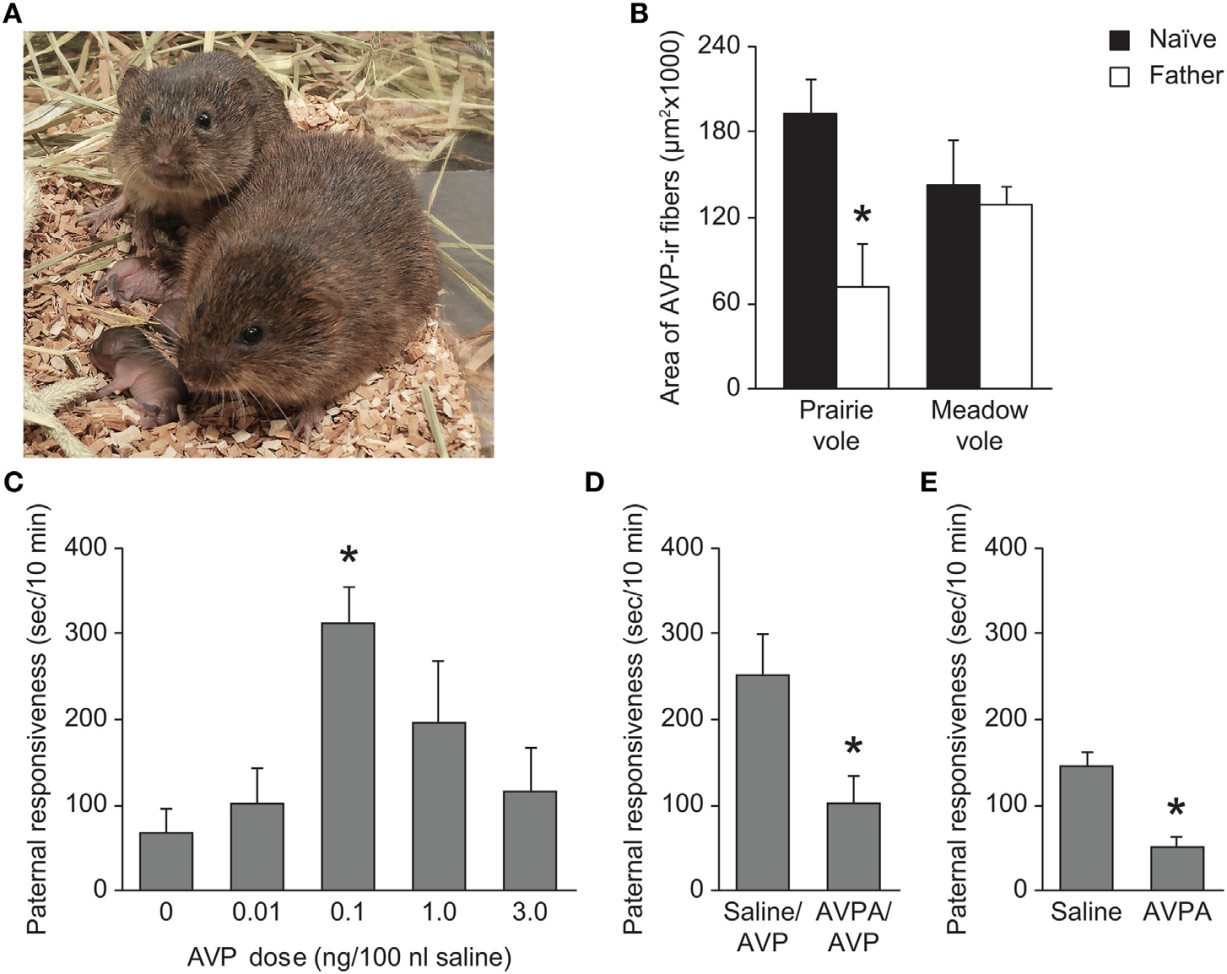
Impact of AVP activity in paternal behavior of prairie voles. **(A)** Prairie voles are a biparental species, and with the exception of nursing, fathers perform all of the same parental behaviors as mothers such as nest building, huddling, and grooming of the young. Photo credit: Charles Badland. **(B)** Prairie vole fathers show a significant decrease in AVP-ir fiber density in the LS relative to sexually naïve prairie voles. This effect is not seen in meadow voles. *p < 0.01 versus naïve voles. **(C)** Administration of AVP into the LS of sexually naïve prairie voles increases paternal responsiveness. *p < 0.0001 versus saline-treated animals. **(D)** Administration of 1 ng AVPA in the LS reduces the AVP-induced (0.1 ng AVP) increase in paternal responsiveness. *p < 0.05 versus saline/AVP-treated animals. **(E)** Administration of 1 ng AVPA reduces baseline levels of paternal responsiveness. *p < 0.001 versus saline-treated controls. AVP, arginine-vasopressin; AVPA, AVPR1a antagonist; LS, lateral septum. Adapted/reproduced from Bamshad et al. (51) with permission from Karger Publishers and Wang et al. (78) with permission from Proceedings of the National Academy of Sciences.

Several studies have established a correlation between AVP-ir fiber expression in the LS and parental behaviors in both male and female prairie voles (Figure 3). AVP-ir fiber density is significantly higher in the LS of male prairie voles than female prairie voles (39, 51). In addition, an increase in AVP-ir fiber density in the LS following estrogen replacement is correlated with an increase in the incidence of maternal behavior in normally infanticidal, ovariectomized females (71). These findings are supported by pharmacological manipulation of AVPR1a in the LS; administration of AVP into the LS promotes parental behavior in naïve male prairie voles, and this effect is prevented by administration of AVPR1a prior to pup exposure (78). Not only does AVP in the LS regulate parental behavior, but AVP fiber density in the LS of male prairie voles is affected by cohabitation and the birth of the first litter as well. Male prairie voles have significantly fewer AVP-ir fibers in the LS than sexually naïve males shortly after mating, as well as 6 days following parturition (51, 53). This decrease in AVP fiber immunoreactivity may reflect an increase of AVP release that has not been recovered. This idea is supported by the finding that AVP mRNA expression in the BNST of male prairie voles is increased as a result of cohabitation with a female (50). These changes in AVP fiber density in the LS and mRNA expression in the BNST are not observed in female prairie voles or meadow voles of either sex (50, 51, 53), suggesting a role of AVP in the LS specifically in paternal behavior.

Although acute antagonism of AVPR1a in the LS leads to a decrease in paternal responsiveness (78), elimination of AVP-ir fibers in the LS as a result of castration does not lead to a decrease in parental behavior (71). This implies that AVP action alone in the LS is not responsible for the modulation of parental behaviors, such that dampened AVP signaling can affect parental behavior transiently while other modulating signals compensate for a prolonged AVP deficit. The onset of paternal behavior may be independent of hormonal regulation, as paternal behavior is spontaneous and observed even in sexually immature juveniles (73). As AVPR1a activity in the LS promotes social recognition in rats and prairie voles (45, 46, 48) and promotes partner preference behavior (40), AVPR1a activation in the LS may induce recognition of a pup as a non-threatening and familiar conspecific that should be cared for. In addition, more recent research has found that only central administration (i.c.v.) of both AVPA and an OTR antagonist together, but not either antagonist alone, is sufficient to reduce parental behavior in naïve male prairie voles (76). This is in contrast to some of the site-specific pharmacology results with AVPA only, which may be due to the quite low concentration of AVPA used in the study, but it does raise the intriguing possibility that AVP and OXT act as redundant and compensatory signals to promote paternal behavior in voles. Moreover, OTR is expressed in the LS (17, 79) and OTR expression in LS is correlated with absence or presence of female alloparental care (74). Therefore, although AVP activity in the LS may promote recognition and reduce infanticide, OTR and AVPR1a in the LS may work in tandem to promote paternal care of the young, though this is only speculation.

## Discussion

The studies discussed above have supported a role of AVP in the formation, expression, and regulation of the pair-bond-related behaviors of the prairie vole. This role of AVP is absent in non-monogamous vole species that do not normally exhibit these behaviors. However, genetic manipulation of AVPR1a expression in specific regions of the brain can induce similar behavior in non-monogamous species, or conversely, eliminate these behaviors in the prairie vole. With this, and site-specific pharmacological manipulation of AVPR1a activity and subsequent behavioral consequences, there is strong evidence supporting a role of AVPR1a distribution and function in the neurobiological basis of social attachment. Central administration of AVP or an AVPR1a selective antagonist regulates behaviors such as partner preference, selective aggression, and paternal care (12, 29, 76). AVP regulation of social behaviors in the prairie vole may be acting through distinct neurocircuits with different roles in relationship formation and maintenance.

The regions in which AVP regulation of partner preference has been characterized, the VP and LS, play important roles in motivation and social recognition in prairie voles (37, 40, 48) and other rodent species (44, 55). In these regions, AVP has a role in regulating affiliative behavior such as partner preference and paternal response. Upregulation of AVPR1a activity in the VP promotes partner preference (37, 42), and reduction of AVPR1a expression in this region drives male prairie voles to prefer an unknown female over the mate (41). In addition, an increase in AVPR1a expression in this region in the non-monogamous meadow vole induces partner preference behavior (38). Upregulation of AVPR1a activity in the LS promotes partner preference (40) and increases paternal behaviors (78). This signaling may have an important role in the formation of affiliative relationships through promotion of recognition of social stimuli, such as a new partner or pup, as conspecifics that should be affiliated with or cared for.

Conversely, AVP signaling in the AH regulates behavior in a manner distinct from VP and LS modulation of positive social relationships. In the AH, AVP signaling regulates selective aggression, and the decision-making process between affiliation toward partners and aggression toward strangers is advantageous in the maintenance of an established bond. Manipulation of AVPR1a activity in the AH not only regulates aggressive behavior in male prairie voles (60, 61), but also appears to regulate the decision to be aggressive or affiliative to a conspecific (63). This regulation of selective aggression occurs as a result of various neurochemicals and brain regions signaling to the AH to either promote or inhibit AVPR1a activity. Overall, burgeoning evidence support a role of AVP in the formation and maintenance of social bonds in prairie vole, possibly through distinct neurocircuitry responsible for social recognition and social decision-making.

Despite this knowledge, some questions remain unanswered. For example, while the *cis*-regulatory element, specifically single-nucleotide polymorphisms and microsatellites, of the prairie vole *avpr1a* gene has been implicated in the regulation of AVPR1a distribution patterns, the specific mechanisms of control of protein expression have been poorly studied. Chromatin remodeling at the *avpr1a* gene has been implicated in the regulation of partner preference behavior. Administration of a histone deacetylase inhibitor into the nucleus accumbens of female prairie voles, but not male prairie voles, upregulates AVPR1a expression and promotes partner preference behavior (80, 81). These findings suggest a sex-specific mechanism of epigenetic modulation of pair bond-related behaviors, and further investigation in this direction could give valuable insight into regulation of social behavior at the transcriptional level. Second, the downstream signaling of AVPR1a and its impact on the regulation of social behavior in the prairie vole has been hardly investigated. One study has examined site-specific induction of phosphoinositol, a second messenger of G_αq_ signaling, following introduction of AVP into the brains of prairie voles and montane voles, and found species differences in regional induction (11). This study, however, did not investigate the impact of site-specific phosphoinositol induction or reduction on social behavior in these species, nor did it investigate induction of other secondary messengers such as cAMP. While AVPR1a has been characterized as a G_αq_-GPCR in hepatocytes (82), it has been suggested to have G_αs_ action in neurons (83). Thus, its signaling cascade should not be assumed.

In addition, one of the shortcomings of not only the studies discussed above, but of prairie vole research in general, is the use of the standard 24-h cohabitation to study neurobiological regulation of pair bond-related behaviors. While this length of cohabitation plus the presence of mating is usually sufficient to induce partner preference and selective aggression, two characteristic behaviors of a pair bond, this model only gives insight into the early stages of pair bond formation. A longer cohabitation period on the scale of days to weeks would allow for investigation of the maintenance of this bond and the behaviors associated with it. Finally, one of the major benefits of the prairie vole model is the similarity of behavior between males and females (29, 30). This allows for investigation of sexual dimorphism in the neurobiological basis of these behaviors. However, much of the research of the impact of AVP on pair bond-related behaviors use male voles and neglect to study any potential impact of AVP in female voles. While it does seem males may be more sensitive to AVP and females to oxytocin (34), there does still appear to be some impact of AVPR1a in the pair bond-related behaviors of female prairie voles (32, 57). Therefore, it is worth including female prairie voles in any study investigating the impact of AVP in social behavior, and if no impact is found, it can be reported so that sexual dimorphism can be noted. In conclusion, while much has been established about the impact of AVP and its action through AVPR1a in the regulation of social behavior, much remains to be discovered. The prairie vole will continue to be a useful model in answering these questions.

## Summary

One of the cognitive mechanisms underlying the formation of a pair bond is theorized to be the learned association between the memory of a partner and reward. Intriguingly, AVP in the LS serves to promote social recognition in rats and prairie voles (45, 46, 48) and partner preference behavior in prairie voles (40). In addition, partner preference formation is facilitated by AVP signaling and AVPR1a expression in the VP (37), a brain region known to play a role in reward and motivation. It is possible that these two neuronal inputs, one regulating recognition and the other reward, converge to promote the selective partner preference. If so, the AVP signal functions as a multimodal neuromodulator of this cognitive mechanism that triggers such bond formation. The AVPR1a distribution in the brain contributes to the social structure of the prairie vole, and genetic variation of the *cis*-regulatory elements of the *avpr1a* gene appear to contribute to these patterns. Thus, as more is learned about the genetic variants that contribute to AVPR1a distribution in the brain, as well as a stronger effort to determine downstream signaling of AVPR1a, this will lead to a coherent framework of the genetic and cellular basis of the AVP system on individual and species level differences in social behavior.

## Author Contributions

MT and AS conceived the review, acquired and critically analyzed the literature, and wrote and critically revised the manuscript.

## Conflict of Interest Statement

The authors declare that the research was conducted in the absence of any commercial or financial relationships that could be construed as a potential conflict of interest.

## Footnotes

^1^While AVP is an important neuromodulator of stress physiology [e.g., functioning as a secondary hypothalamic-pituitary-adrenal (HPA) axis secretagogue], it has yield to be determined the role that AVP serves in separation distress, consoling behavior, or social buffering.

## References

[1] Kleiman DG. Monogamy in mammals. Q Rev Biol (1977) 52:39–69. 10.1086/409721 857268

[2] Getz LL Carter CS Gavish L The mating system of the prairie vole, *Microtus ochrogaster*: field and laboratory evidence for pair-bonding. Behav Ecol Sociobiol (1981) 8:189–94. 10.1007/BF00299829

[3] Dewsbury DA. The comparative psychology of monogamy. Nebr Symp Motiv (1987) 35:1–50. 3332030

[4] Shapiro LE Dewsbury DA. Differences in affiliative behavior, pair bonding, and vaginal cytology in two species of vole (*Microtus ochrogaster* and *M. montanus*). J Comp Psychol (1990) 104:268–74. 10.1037/0735-7036.104.3.268 2225765

[5] Gruder-Adams S Getz LL Comparison of the mating system and paternal behavior in *Microtus ochrogaster* and *Microtus pennsylvanicus*. J Mammal (1985) 66:165–7. 10.2307/1380976

[6] Bielsky IF Hu SB Szegda KL Westphal H Young LJ. Profound impairment in social recognition and reduction in anxiety-like behavior in vasopressin V1a receptor knockout mice. Neuropsychopharmacology (2004) 29:483–93. 10.1038/sj.npp.1300360 14647484

[7] Egashira N Tanoue A Matsuda T Koushi E Harada S Takano Y Impaired social interaction and reduced anxiety-related behavior in vasopressin V1a receptor knockout mice. Behav Brain Res (2007) 178:123–7. 10.1016/j.bbr.2006.12.009 17227684

[8] Fodor A Barsvari B Aliczki M Balogh Z Zelena D Goldberg SR The effects of vasopressin deficiency on aggression and impulsiveness in male and female rats. Psychoneuroendocrinology (2014) 47:141–50. 10.1016/j.psyneuen.2014.05.010 25001964

[9] Bosch OJ Neumann ID. Vasopressin released within the central amygdala promotes maternal aggression. Eur J Neurosci (2010) 31:883–91. 10.1111/j.1460-9568.2010.07115.x 20374286

[10] Bayerl DS Honig JN Bosch OJ Vasopressin V1a, but not V1b, receptors within the PVN of lactating rats mediate maternal care and anxiety-related behaviour. Behav Brain Res (2016) 305:18–22. 10.1016/j.bbr.2016.02.020 26909846

[11] Insel TR Wang ZX Ferris CF. Patterns of brain vasopressin receptor distribution associated with social organization in microtine rodents. J Neurosci (1994) 14:5381–92. 8083743 10.1523/JNEUROSCI.14-09-05381.1994 PMC6577077

[12] Young LJ Winslow JT Nilsen R Insel TR. Species differences in V1a receptor gene expression in monogamous and nonmonogamous voles: behavioral consequences. Behav Neurosci (1997) 111:599–605. 10.1037/0735-7044.111.3.599 9189274

[13] Wang Z Young LJ Liu Y Insel TR. Species differences in vasopressin receptor binding are evident early in development: comparative anatomic studies in prairie and montane voles. J Comp Neurol (1997) 378:535–46. 10.1002/(SICI)1096-9861(19970224)378:4<535::AID-CNE8>3.0.CO;2-3 9034909

[14] Smeltzer MD Curtis JT Aragona BJ Wang Z. Dopamine, oxytocin, and vasopressin receptor binding in the medial prefrontal cortex of monogamous and promiscuous voles. Neurosci Lett (2006) 394:146–51. 10.1016/j.neulet.2005.10.019 16289323

[15] Phelps SM Young LJ. Extraordinary diversity in vasopressin (V1a) receptor distributions among wild prairie voles (*Microtus ochrogaster*): patterns of variation and covariation. J Comp Neurol (2003) 466:564–76. 10.1002/cne.10902 14566950

[16] Cushing BS Okorie U Young LJ. The effects of neonatal castration on the subsequent behavioural response to centrally administered arginine vasopressin and the expression of V1a receptors in adult male prairie voles. J Neuroendocrinol (2003) 15:1021–6. 10.1046/j.1365-2826.2003.01097.x 14622431

[17] Lim MM Murphy AZ Young LJ. Ventral striatopallidal oxytocin and vasopressin V1a receptors in the monogamous prairie vole (*Microtus ochrogaster*). J Comp Neurol (2004) 468:555–70. 10.1002/cne.10973 14689486

[18] Young LJ Nilsen R Waymire KG MacGregor GR Insel TR. Increased affiliative response to vasopressin in mice expressing the V1a receptor from a monogamous vole. Nature (1999) 400:766–8. 10.1038/23475 10466725

[19] Ophir AG Campbell P Hanna K Phelps SM. Field tests of cis-regulatory variation at the prairie vole avpr1a locus: association with V1aR abundance but not sexual or social fidelity. Horm Behav (2008) 54:694–702. 10.1016/j.yhbeh.2008.07.009 18722379

[20] Hammock EAD Lim MM Nair HP Young LJ. Association of vasopressin 1a receptor levels with a regulatory microsatellite and behavior. Genes Brain Behav (2005) 4:289–301. 10.1111/j.1601-183X.2005.00119.x 16011575

[21] Hammock EAD Young LJ. Microsatellite instability generates diversity in brain and sociobehavioral traits. Science (2005) 308:1630–4. 10.1126/science.1111427 15947188

[22] Mabry KE Streatfeild CA Keane B Solomon NG. Avpr1a length polymorphism is not associated with either social or genetic monogamy in free-living prairie voles. Anim Behav (2011) 81:11–8. 10.1016/j.anbehav.2010.09.021 21442019 PMC3063090

[23] Okhovat M Berrio A Wallace G Ophir AG Phelps SM. Sexual fidelity trade-offs promote regulatory variation in the prairie vole brain. Science (2015) 350:1371–4. 10.1126/science.aac5791 26659055

[24] Ophir AG Wolff JO Phelps SM. Variation in neural V1aR predicts sexual fidelity and space use among male prairie voles in semi-natural settings. Proc Natl Acad Sci U S A (2008) 105:1249–54. 10.1073/pnas.0709116105 18212120 PMC2234124

[25] Solomon NG Richmond AR Harding PA Fries A Jacquemin S Schaefer RL Polymorphism at the avpr1a locus in male prairie voles correlated with genetic but not social monogamy in field populations. Mol Ecol (2009) 18:4680–95. 10.1111/j.1365-294X.2009.04361.x 19821904

[26] Donaldson ZR Young LJ. The relative contribution of proximal 5’ flanking sequence and microsatellite variation on brain vasopressin 1a receptor (Avpr1a) gene expression and behavior. PLoS Genet (2013) 9:e1003729. 10.1371/journal.pgen.1003729 24009523 PMC3757045

[27] Okhovat M Chen IC Dehghani Z Zheng DJ Ikpatt JE Momoh H Genetic variation in the developmental regulation of cortical avpr1a among prairie voles. Genes Brain Behav (2017). 10.1111/gbb.12396 28589689

[28] Okhovat M Maguire SM Phelps SM. Methylation of avpr1a in the cortex of wild prairie voles: effects of CpG position and polymorphism. R Soc Open Sci (2017) 4:160646. 10.1098/rsos.160646 28280564 PMC5319330

[29] Winslow JT Hastings N Carter CS Harbaugh CR Insel TR. A role for central vasopressin in pair bonding in monogamous prairie voles. Nature (1993) 365:545–8. 10.1038/365545a0 8413608

[30] Williams JR Catania KC Carter CS. Development of partner preferences in female prairie voles (*Microtus ochrogaster*): the role of social and sexual experience. Horm Behav (1992) 26:339–49. 10.1016/0018-506X(92)90004-F 1398553

[31] Insel TR Preston S Winslow JT. Mating in the monogamous male: behavioral consequences. Physiol Behav (1995) 57:615–27. 10.1016/0031-9384(94)00362-9 7777594

[32] Cho MM DeVries AC Williams JR Carter CS. The effects of oxytocin and vasopressin on partner preferences in male and female prairie voles (*Microtus ochrogaster*). Behav Neurosci (1999) 113:1071–9. 10.1037/0735-7044.113.5.1071 10571489

[33] Sun P Smith AS Lei K Liu Y Wang Z. Breaking bonds in male prairie vole: long-term effects on emotional and social behavior, physiology, and neurochemistry. Behav Brain Res (2014) 265:22–31. 10.1016/j.bbr.2014.02.016 24561258 PMC3983777

[34] Insel TR Hulihan TJ. A gender-specific mechanism for pair bonding: oxytocin and partner preference formation in monogamous voles. Behav Neurosci (1995) 109:782–9. 10.1037/0735-7044.109.4.782 7576222

[35] Curtis JT. Does fertility trump monogamy? Anim Behav (2010) 80:319–28. 10.1016/j.anbehav.2010.05.014 20823948 PMC2930788

[36] Donaldson ZR Spiegel L Young LJ. Central vasopressin V1a receptor activation is independently necessary for both partner preference formation and expression in socially monogamous male prairie voles. Behav Neurosci (2010) 124:159–63. 10.1037/a0018094 20141291 PMC2846693

[37] Pitkow LJ Sharer CA Ren X Insel TR Terwilliger EF Young LJ. Facilitation of affiliation and pair-bond formation by vasopressin receptor gene transfer into the ventral forebrain of a monogamous vole. J Neurosci (2001) 21:7392–6. 11549749 10.1523/JNEUROSCI.21-18-07392.2001 PMC6762997

[38] Lim MM Wang Z Olazabal DE Ren X Terwilliger EF Young LJ. Enhanced partner preference in a promiscuous species by manipulating the expression of a single gene. Nature (2004) 429:754–7. 10.1038/nature02539 15201909

[39] Wang Z Species differences in the vasopressin-immunoreactive pathways in the bed nucleus of the stria terminalis and medial amygdaloid nucleus in prairie voles (*Microtus ochrogaster*) and meadow voles (*Microtus pennsylvanicus*). Behav Neurosci (1995) 109:305–11. 10.1037/0735-7044.109.2.305 7619320

[40] Liu Y Curtis JT Wang Z. Vasopressin in the lateral septum regulates pair bond formation in male prairie voles (*Microtus ochrogaster*). Behav Neurosci (2001) 115:910–9. 10.1037/0735-7044.115.4.910 11508730

[41] Barrett CE Keebaugh AC Ahern TH Bass CE Terwilliger EF Young LJ. Variation in vasopressin receptor (Avpr1a) expression creates diversity in behaviors related to monogamy in prairie voles. Horm Behav (2013) 63:518–26. 10.1016/j.yhbeh.2013.01.005 23370363 PMC3602142

[42] Lim MM Young LJ. Vasopressin-dependent neural circuits underlying pair bond formation in the monogamous prairie vole. Neuroscience (2004) 125:35–45. 10.1016/j.neuroscience.2003.12.008 15051143

[43] Bielsky IF Hu S-B Ren X Terwilliger EF Young LJ. The V1a vasopressin receptor is necessary and sufficient for normal social recognition: a gene replacement study. Neuron (2005) 47:503–13. 10.1016/j.neuron.2005.06.031 16102534

[44] Lukas M Bredewold R Landgraf R Neumann ID Veenema AH. Early life stress impairs social recognition due to a blunted response of vasopressin release within the septum of adult male rats. Psychoneuroendocrinology (2011) 36:843–53. 10.1016/j.psyneuen.2010.11.007 21185124

[45] Engelmann M Landgraf R. Microdialysis administration of vasopressin into the septum improves social recognition in Brattleboro rats. Physiol Behav (1994) 55:145–9. 10.1016/0031-9384(94)90022-1 8140159

[46] Everts HG Koolhaas JM. Lateral septal vasopressin in rats: role in social and object recognition? Brain Res (1997) 760:1–7. 10.1016/S0006-8993(97)00269-2 9237511

[47] Landgraf R Frank E Aldag JM Neumann ID Sharer CA Ren X Viral vector-mediated gene transfer of the vole V1a vasopressin receptor in the rat septum: improved social discrimination and active social behaviour. Eur J Neurosci (2003) 18:403–11. 10.1046/j.1460-9568.2003.02750.x 12887422

[48] Ophir AG Zheng D-J Eans S Phelps SM Social investigation in a memory task relates to natural variation in septal expression of oxytocin receptor and vasopressin receptor 1a in prairie voles (*Microtus ochrogaster*). Behav Neurosci (2009) 123:979–91. 10.1037/a0016663 19824764

[49] DeVries GJ Buijs RM Van Leeuwen FW Caffe AR Swaab DF. The vasopressinergic innervation of the brain in normal and castrated rats. J Comp Neurol (1985) 233:236–54. 10.1002/cne.902330206 3882778

[50] Wang Z Smith W Major DE De Vries GJ Sex and species differences in the effects of cohabitation on vasopressin messenger RNA expression in the bed nucleus of the stria terminalis in prairie voles (*Microtus ochrogaster*) and meadow voles (*Microtus pennsylvanicus*). Brain Res (1994) 650:212–8. 10.1016/0006-8993(94)91784-1 7953686

[51] Bamshad M Novak MA De Vries GJ. Sex and species differences in the vasopressin innervation of sexually naive and parental prairie voles, *Microtus ochrogaster* and meadow voles, *Microtus pennsylvanicus*. J Neuroendocrinol (1993) 5:247–55. 10.1111/j.1365-2826.1993tb00480.x 8319000

[52] Wang Z Zhou L Hulihan TJ Insel TR. Immunoreactivity of central vasopressin and oxytocin pathways in microtine rodents: a quantitative comparative study. J Comp Neurol (1996) 366:726–37. 10.1002/(SICI)1096-9861(19960318)366:4<726::AID-CNE11>3.0.CO;2-D 8833119

[53] Bamshad M Novak MA de Vries GJ Cohabitation alters vasopressin innervation and paternal behavior in prairie voles (*Microtus ochrogaster*). Physiol Behav (1994) 56:751–8. 10.1016/0031-9384(94)90238-0 7800744

[54] Cromwell HC Berridge KC. Where does damage lead to enhanced food aversion: the ventral pallidum/substantia innominata or lateral hypothalamus? Brain Res (1993) 624:1–10. 10.1016/0006-8993(93)90053-P 8252379

[55] Farrar AM Font L Pereira M Mingote S Bunce JG Chrobak JJ Forebrain circuitry involved in effort-related choice: injections of the GABA(A) agonist muscimol into ventral pallidum alter response allocation in food-seeking behavior. Neuroscience (2008) 152:321–30. 10.1016/j.neuroscience.2007.12.034 18272291 PMC2668809

[56] Dallimore JE Mickiewicz AL Napier TC. Intra-ventral pallidal glutamate antagonists block expression of morphine-induced place preference. Behav Neurosci (2006) 120:1103–14. 10.1037/0735-7044.120.5.1103 17014261

[57] Zheng D-J Larsson B Phelps SM Ophir AG. Female alternative mating tactics, reproductive success and nonapeptide receptor expression in the social decision-making network. Behav Brain Res (2013) 246:139–47. 10.1016/j.bbr.2013.02.024 23500897 PMC3633648

[58] Ophir AG Sorochman G Evans BL Prounis GS. Stability and dynamics of forebrain vasopressin receptor and oxytocin receptor during pregnancy in prairie voles. J Neuroendocrinol (2013) 25:719–28. 10.1111/jne.12049 23656585 PMC3716852

[59] Wang Z Hulihan TJ Insel TR. Sexual and social experience is associated with different patterns of behavior and neural activation in male prairie voles. Brain Res (1997) 767:321–32. 10.1007/PL00005640 9367264

[60] Gobrogge KL Liu Y Jia X Wang Z. Anterior hypothalamic neural activation and neurochemical associations with aggression in pair-bonded male prairie voles. J Comp Neurol (2007) 502:1109–22. 10.1002/cne.21364 17444499

[61] Gobrogge KL Liu Y Young LJ Wang Z Anterior hypothalamic vasopressin regulates pair-bonding and drug-induced aggression in a monogamous rodent. Proc Natl Acad Sci U S A (2009) 106:19144–9. 10.1073/pnas.0908620106 19858480 PMC2776424

[62] Blondel DV Phelps SM Effects of acute corticosterone treatment on male prairie voles (*Microtus ochrogaster*): territorial aggression does not accompany induced social preference. J Comp Psychol (2016) 130:400–6. 10.1037/com0000048 27841456 PMC5131865

[63] Gobrogge KL Jia X Liu Y Wang Z. Neurochemical mediation of affiliation and aggression associated with pair-bonding. Biol Psychiatry (2017) 81:231–42. 10.1016/j.biopsych.2016.02.013 27129413 PMC4992658

[64] Carter CS DeVries AC Getz LL. Physiological substrates of mammalian monogamy: the prairie vole model. Neurosci Biobehav Rev (1995) 19:303–14. 10.1016/0149-7634(94)00070-H 7630584

[65] Aragona BJ Liu Y Yu YJ Curtis JT Detwiler JM Insel TR Nucleus accumbens dopamine differentially mediates the formation and maintenance of monogamous pair bonds. Nat Neurosci (2006) 9:133–9. 10.1038/nn1613 16327783

[66] Bowler CM Cushing BS Carter CS. Social factors regulate female-female aggression and affiliation in prairie voles. Physiol Behav (2002) 76:559–66. 10.1016/S0031-9384(02)00755-2 12126993

[67] Firestone KB Thompson KV Carter CS Female-female interactions and social stress in praine voles. Behav Neural Biol (1991) 55:31–41. 10.1016/0163-1047(91)80125-X 1996946

[68] Ferris CF Melloni RH Jr Koppel G Perry KW Fuller RW Delville Y. Vasopressin/serotonin interactions in the anterior hypothalamus control aggressive behavior in golden hamsters. J Neurosci (1997) 17:4331–40. 9151749 10.1523/JNEUROSCI.17-11-04331.1997 PMC6573530

[69] Gutzler SJ Karom M Erwin WD Albers HE Arginine-vasopressin and the regulation of aggression in female Syrian hamsters (*Mesocricetus auratus*). Eur J Neurosci (2010) 31:1655–63. 10.1111/j.1460-9568.2010.07190.x 20525078

[70] Lonstein JS De Vries GJ. Comparison of the parental behavior of pair-bonded female and male prairie voles (*Microtus ochrogaster*). Physiol Behav (1999) 66:33–40. 10.1016/S0031-9384(98)00270-4 10222470

[71] Lonstein JS De Vries GJ. Sex differences in the parental behaviour of adult virgin prairie voles: independence from gonadal hormones and vasopressin. J Neuroendocrinol (1999) 11:441–9. 10.1046/j.1365-2826.1999.00361.x 10336725

[72] Lonstein JS De Vries GJ. Influence of gonadal hormones on the development of parental behavior in adult virgin prairie voles (*Microtus ochrogaster*). Behav Brain Res (2000) 114:79–87. 10.1016/S0166-4328(00)00192-3 10996049

[73] Stone AI Mathieu D Griffin L Bales KL. Alloparenting experience affects future parental behavior and reproductive success in prairie voles (*Microtus ochrogaster*). Behav Processes (2010) 83:8–15. 10.1016/j.beproc.2009.08.008 19732810 PMC2814911

[74] Olazabal DE Young LJ. Species and individual differences in juvenile female alloparental care are associated with oxytocin receptor density in the striatum and the lateral septum. Horm Behav (2006) 49:681–7. 10.1016/j.yhbeh.2005.12.010 16442534

[75] Lonstein JS De Vries GJ. Social influences on parental and nonparental responses toward pups in virgin female prairie voles (*Microtus ochrogaster*). J Comp Psychol (2001) 115:53–61. 10.1037/0735-7036.115.1.53 11334219

[76] Bales KL Kim AJ Lewis-Reese AD Sue Carter C. Both oxytocin and vasopressin may influence alloparental behavior in male prairie voles. Horm Behav (2004) 45:354–61. 10.1016/j.yhbeh.2004.01.004 15109910

[77] Parker KJ Lee TM. Central vasopressin administration regulates the onset of facultative paternal behavior in *Microtus pennsylvanicus* (meadow voles). Horm Behav (2001) 39:285–94. 10.1006/hbeh.2001.1655 11374914

[78] Wang Z Ferris CF De Vries GJ. Role of septal vasopressin innervation in paternal behavior in prairie voles (*Microtus ochrogaster*). Proc Natl Acad Sci U S A (1994) 91:400–4. 10.1073/pnas.91.1.400 8278401 PMC42955

[79] Wang Z Young LJ. Ontogeny of oxytocin and vasopressin receptor binding in the lateral septum in prairie and montane voles. Brain Res Dev Brain Res (1997) 104:191–5. 10.1016/S0165-3806(97)00138-7 9466721

[80] Wang H Duclot F Liu Y Wang Z Kabbaj M. Histone deacetylase inhibitors facilitate partner preference formation in female prairie voles. Nat Neurosci (2013) 16:919–24. 10.1038/nn.3420 23727821 PMC3703824

[81] Duclot F Wang H Youssef C Liu Y Wang Z Kabbaj M. Trichostatin A (TSA) facilitates formation of partner preference in male prairie voles (*Microtus ochrogaster*). Horm Behav (2016) 81:68–73. 10.1016/j.yhbeh.2016.04.001 27074037 PMC4893910

[82] Thibonnier M Auzan C Madhun Z Wilkins P Berti-Mattera L Clauser E. Molecular cloning, sequencing, and functional expression of a cDNA encoding the human V1a vasopressin receptor. J Biol Chem (1994) 269:3304–10. 8106369

[83] Wrobel LJ Dupre A Raggenbass M. Excitatory action of vasopressin in the brain of the rat: role of cAMP signaling. Neuroscience (2011) 172:177–86. 10.1016/j.neuroscience.2010.10.006 20933582

